# Building biosecurity for synthetic biology

**DOI:** 10.15252/msb.20209723

**Published:** 2020-07-21

**Authors:** Benjamin D Trump, SE Galaitsi, Evan Appleton, Diederik A Bleijs, Marie‐Valentine Florin, Jimmy D Gollihar, R Alexander Hamilton, Todd Kuiken, Filippa Lentzos, Ruth Mampuys, Myriam Merad, Tatyana Novossiolova, Kenneth Oye, Edward Perkins, Natàlia Garcia‐Reyero, Catherine Rhodes, Igor Linkov

**Affiliations:** ^1^ US Army Corps of Engineers, Engineer Research and Development Center Vicksburg MS USA; ^2^ Harvard University Cambridge MA USA; ^3^ Biosecurity Office Netherlands National Institute for Public Health and the Environment (RIVM) Bilthoven The Netherlands; ^4^ International Risk Governance Center École Polytechnique Fédérale de Lausanne Lausanne Switzerland; ^5^ US Army Research Laboratory (ARL) Adelphi MD USA; ^6^ United Nations Interregional Crime and Justice Research Institute (UNICRI) Turin Italy; ^7^ Genetic Engineering & Society Center at North Carolina State University Raleigh NC USA; ^8^ King's College London London UK; ^9^ Netherlands Commission on Genetic Modification (COGEM) Bilthoven The Netherlands; ^10^ French National Centre for Scientific Research Paris France; ^11^ Center for the Study of Democracy Sofia Bulgaria; ^12^ Massachusetts Institute of Technology Cambridge MA USA; ^13^ University of Cambridge Cambridge UK

**Keywords:** Synthetic Biology & Biotechnology, S&S: Ethics

## Abstract

The fast‐paced field of synthetic biology is fundamentally changing the global biosecurity framework. Current biosecurity regulations and strategies are based on previous governance paradigms for pathogen‐oriented security, recombinant DNA research, and broader concerns related to genetically modified organisms (GMOs). Many scholarly discussions and biosecurity practitioners are therefore concerned that synthetic biology outpaces established biosafety and biosecurity measures to prevent deliberate and malicious or inadvertent and accidental misuse of synthetic biology's processes or products. This commentary proposes three strategies to improve biosecurity: Security must be treated as an investment in the future applicability of the technology; social scientists and policy makers should be engaged early in technology development and forecasting; and coordination among global stakeholders is necessary to ensure acceptable levels of risk.

## Graphical abstract

Biosecurity policies and practices must be updated to accommodate the novel challenges associated with synthetic biology and to maximize technological benefits while minimizing its dual‐use potential. This Commentary proposes three strategies to improve biosecurity.

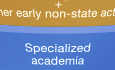

All technology has dual‐use aspects: It can be used for beneficial and harmful purposes. The Internet is a source of limitless information and interaction but it also enables much criminal behavior under the guise of anonymity. Similarly, synthetic biology (SB) has great potential for beneficial and valuable applications and products but could also be misused to harm humans or the environment. Governance regimes must therefore balance mitigating the risk of misuse with supporting opportunities for innovation and development. However, biosecurity efforts remain mired in uncertainty about the capabilities of SB and its practitioners’ motivations in the growing number of contexts in which it is applied. Two decades into the 21^st^ century, governments are still imposing old rules on a new technology, an insufficient strategy to provide security in the future.

Though it lacks a universal definition, SB has been described as “designing and constructing biological modules, biological systems, and biological machines or, re‐design of existing biological systems for useful purposes” (Nakano *et al*, 2013). The Engineering Biology Research Consortium (EBRC) puts SB in an evolutionary context that “builds on the advances in molecular, cell, and systems biology” to design and construct genetic circuits, metabolic pathways, or other constructs to address defined objectives. Building an effective biosecurity strategy that covers these SB design approaches and technologies requires understanding the novel threats that these technologies create, along with the structural vulnerabilities products stemming from these technologies can exploit and the likely causes of inadequate biosecurity practices. New concerns arise from SB's broad scope, wider availability, complexity, and uncertainty over current and future capabilities. One very critical technology is gene editing to precisely modify genomes. One of its applications, gene drive, has raised particular concerns as it can quickly propagate a specific suite of genes or alleles through a population by circumventing Mendelian inheritance and thereby increasing the probability that these genes are passed on to offspring.

Gene editing has enormous potential for improving human health, agriculture, and the environment, but it can also cause substantial and irreversible harms. Such harms might include the uncontrolled diffusion of gene‐edited material in the environment, off‐target effects from genome editing, or the disruption of ecologies with genetically altered organisms, especially with engineered gene drive systems. Harms may also arise through the deliberate use of these techniques to target humans and/or the environment. Such intentional misuse of SB techniques requires two circumstances: the availability of techniques and know‐how that could be exploited for irresponsible or nefarious purposes (“information hazard”); and the ability to use such knowledge and tools to generate and disseminate harmful engineered organisms.

Such knowledge includes the publication of a method for synthesizing horsepox; some critics argue that this information might reasonably enable a nefarious actor to reconstitute smallpox or to synthetize other viruses. Additionally, the widely publicized sequence and recreation of the 1918 Spanish Influenza virus, which killed some 50 million people at the close of the First World War (Evans & Selgelid, 2015), could enable other actors to cause harm. Even non‐pathogenic approaches have been described as dual‐use research, ranging from the disruption of local ecologies via gene drives to the manipulation or destruction of inorganic materials through engineered bacteria.

## Potential misusers

These and other cases show that interested actors or parties can acquire information and apply existing tools for advanced genetic engineering with limited to no oversight. Those who may choose to misuse SB could possess a broad diversity of backgrounds, motivations, strategic goals, and resources. From a top‐down perspective, classical global biosecurity has focused on state actors with the scientific and technological know‐how to pursue offensive biological research, including infamous examples such as Imperial Japan's Unit 731 during World War II, as well as postwar biological weapons programs in the UK, the USSR, and the USA. Typically, such offensive biological weapons capabilities were framed as “first strike” options or avenues to destabilize enemy ground forces that would not easily be deterred by conventional ordnance, such as the Hussein Government's development of bioweapons during the Iran‐Iraq War via anthrax, botulinum toxin, and aflatoxin. In each instance, states developed scientific and materiel capability to construct, package, and deploy biological weapons for strategic measures.

More recently, the pursuit of biological weapons has extended to non‐state organizations. For example, al‐Qaeda (anthrax) and Aum Shinrikyo (anthrax, botulinum toxin) both demonstrated an interest in bioweapons but with very limited success given the technological constraints at the time. However, even individuals or small cells have increased in number and destructive potential. Among the most infamous examples is Bruce Edwards Ivins, who according to the US Department of Justice, leveraged institutional resources as the sole actor responsible for the 2001 deployment of anthrax in letters to Congress and the media. Other actors could be disgruntled employees of sophisticated scientific laboratories or vengeful academics. As advances in genetic engineering become more accessible to private persons, a question remains regarding the rate‐limiting steps (e.g., technical knowledge or inspiration) for such individual actors or small groups to pursue more sophisticated biological weapons.

**Figure 1:**
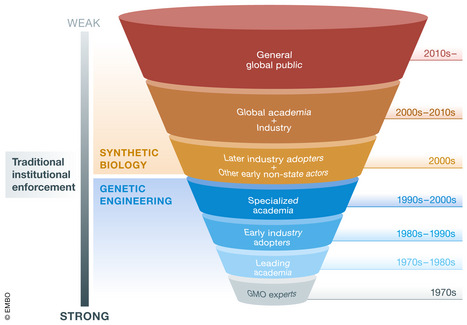
**Increasing number of global users able to access genetic engineering (blue) and synthetic biology (orange) technologies over time**.

Currently, significant barriers remain for independent actors to access critical equipment and materials, but oversight organizations are not prepared for a future when intangible transfers reduce or overcome these obstacles. In 1975, the US National Institutes of Health (NIH) established compliance measures for genome engineering that were enforced through funding restrictions; however, much SB research now operates without NIH funding, approval or even awareness, and NIH does not oversee research in other countries. Today, the financial costs, time limitations, and skill requirements needed to use SB tools have scaled down to become even more broadly accessible. Furthermore, the requisite baseline knowledge will diminish over time as SB processes become more streamlined. While such broad access to sophisticated genetic engineering knowledge and equipment can accelerate scientific breakthroughs, it also places the responsibility of biosecurity on a near‐infinite number of unsupervised actors across the globe (Fig 1). Indeed, in 2018, the States Parties to the Biological Weapons Convention (BWC) noted that access to technologies such as gene editing, gene drives, and gene synthesis is increasingly being conferred to actors with limited or no oversight from established industry or governmental organizations, raising concerns about potential violations of the BWC.

## Lack of oversight

It is helpful to forecast and understand looming threats and potential mitigation strategies at various scales, but international treaties are not structured to oversee bottom‐up efforts related to SB below the national scale. One response may be more engagement by overseeing agencies such as the NIH. Another option is the Responsible Research and Innovation (RRI) approach to assess societal implications of emerging research to better align processes and expected outcomes with the needs and values of society. Where top‐down governance proves insufficient, other actors such as universities, non‐profits, and companies will need to act as gatekeepers and watchdogs to protect against nefarious actors. Top‐down governance may then support such initiatives, which will require harmonization and communication at the international level.

Long‐standing biosecurity policy practices appear to have gaps in biosecurity oversight for SB. These policies include the framing of security as a cost or undesirable expense; the siloing of scholarship and practice across disciplinary domains and between academia, government, industry, and civil society; and the narrow framing of security issues that ignore technology developments. Each of these concerns could be addressed by policy solutions that both support technological development and mitigate security threats while facilitating public engagement in SB and investment in its products. These policies must be scalable, transferrable, and adaptable to incorporate emerging technical and social challenges.

## Security must be an investment, not a cost

Investment implies allocating resources with the expectation of greater gains in the future. To incentivize investments in biosecurity, the entity that provides the initial resources must therefore have a share in future benefits. At present, however, biosecurity is framed as an obligation for individual scientists, organizations, and companies to use institutional funds to comply with unstated and often ambiguous needs for general security. This is an unstable balance of costs and benefits, and thus, few institutions prefer to minimize expenditures associated with fulfilling basic oversight requirements (Gillum *et al*, 2018). Yet, the best argument in favor of investing in biosecurity is that SB's development requires public acceptance, which remains tentative at present (Oliver, 2018). Such acceptance could drop precipitously if the public is inadvertently exposed to harm as a result of a lack of oversight. Biosecurity therefore requires an approach that incentivizes managers to keep abreast of risks and concerns. Biosecurity can signal to the general public that SB products have been appropriately screened to assure beneficial uses. The US Nuclear Regulatory Commission (nrc.gov) performs this for research and test reactors, mostly in universities or colleges, but there is no analogous commission for biosecurity.

The members of the International Gene Synthesis Consortium (IGSC)—which are most of the DNA synthesis companies—devote company resources to screening customers and their requests for potential security issues: This is in the best interests of the companies, despite the fact that no legal mandate requires them to do so. Similarly, in January 2020, the Nuclear Threat Initiative (NTI) and the World Economic Forum recommended in a report the establishment of a Technical Consortium to develop a common DNA sequence screening mechanism, following up on work and conclusions by the IGSC. Across such efforts, Industry has realized that companies stand to gain from aligning themselves with the risk aversion of the public and to invest accordingly in security. This was the lesson for the chemical industry that initially lobbied against the 1925 Geneva Protocol against chemical weapons and prevented its ratification for 50 years in the USA (Tucker, 2007). Today's chemical industry is a strong advocate of chemical arms control (e.g., The Responsible Care Programme).

## Bridges are needed between biosecurity experts, social scientists, and practitioners

Many emerging technologies develop out of sight of social scientists and policy commentators (Linkov *et al*, 2018). Institutional incentives to advance science and technology usually do not create opportunities for inquiry and discussion between developers, risk assessors, ethicists, and policy analysts at the early stages of research. As a result, social science discussions, especially those that the public may relate to such as concerns related to ethics, morals, and risk to health, are often relegated to an afterthought and isolated within institutions or organizations. To address the problem, the RRI programs in the UK and the EU involve experts from diverse fields to assess scientific advancement with the aim of mitigating risk, upholding core morals and values, and achieving research commodification in equitable and sustainable means. Measures such as RRI are not intended to block research or publication of results, but to reduce downstream harms that might place developers, companies, and governments potentially responsible for expensive cleanup and/or insurance efforts. More social inquiry alongside significant funding for SB will improve deliberation into potential biosecurity threats and reduce the potential for unexpected dual‐use publications or developments.

The lack of transparency within the process of technical development removes an essential opportunity to consider whether an idea or goal presents a biosecurity hazard that broader society will not condone. A recent example includes Dr. Jiankui He's work to produce the world's first genome‐edited babies in late 2018. His experiments were widely condemned by leading biologists around the world, yet the small circle of people and institutions that engaged with his experiment while it was underway did nothing to stop him (Cohen, 2019). Broader engagement may have shifted or even halted Dr. He's work to better align with global norms and expectations for human experimentation.

## The future of biosecurity must be a collective global effort

Neither “synthetic biology” nor “biosecurity” has a universally accepted definition, leaving states and organizations to include or exclude lines of research depending on their risk tolerance or incentives in pursuing specific goals. Though conventions such as the BWC and Chemical Weapons Convention (CWC) provide common ground related to weaponry, the ethics and practices that support their objectives are not necessarily taught or enforced within all institutions.

Institutional, political, and economic influences shape local attitudes toward the perception, management, and communication of risk from emerging technologies, requiring that biosecurity measures are tailored to different national and institutional contexts. However, biosecurity equally represents a global public good, requiring international dialogue and collaboration to achieve minimum biosecurity standards. Where some governments or industries may adopt a precautionary approach to manage uncertain biological threats, others may be more risk tolerant and thereby more vulnerable to certain threats. Countries may also avoid disclosing information about their activities or committing themselves to any restrictions on behaviors.

Challenges stemming from diverging practices of SB biosecurity governance are exacerbated by the increasingly globalized, dispersed, and distributed nature of the technology and its research. Advanced biological research is no longer dominated by the Western world, and this may require different approaches to or priorities for biosecurity. Russia's Federal Research Programme for Genetic Technologies Development for 2019–2027 intends to “implement a comprehensive solution to the task of the accelerated development of genetic technologies, including genetic editing…” Saudi Arabia is funding research to develop microbial cell factories to produce fuels and chemicals, while Singapore is investing considerable resources into life and environmental sciences research. The Chinese Academy of Sciences is establishing an Institute of Synthetic Biology, which is tasked with the dual responsibilities of fostering roadmaps for future development while establishing safety and security norms for researchers at Chinese institutions. There are no top‐down efforts beyond existing mechanisms like the BWC or the CWC to standardize global governance and usage of SB, and bottom‐up efforts are not coordinated in their reach or messaging.

Newcomers to SB may have differing tolerances and understandings of risk than more experienced technology developers. The implications, though vast, can be grouped into two general areas. One includes diverging safety and security practices at various points of an international supply chain that forms the backbone of an increasingly globalized economy. Another includes the potential for small‐scale experiments to escape national biosecurity control and spill across political boundaries. While one country may find the environmental risk of a particular SB application acceptable, its spread across borders into another country may disrupt those local ecologies or expose vulnerable human, animal, and plant populations to irreversible consequences. The nature of certain SB applications, in particular gene drive, makes it impossible for risk‐averse countries to wholly quarantine themselves from another country's decisions. This is also an issue of equity: Risk‐tolerant countries will reap the rewards when beneficial technologies emerge, while risk‐averse countries may bear their neighbor's risks without any means to capture potential rewards.

An environment of competing and incongruent risk architectures causes individual states, organizations, or industries to arrive at differing definitions of security threats or acceptable levels of loss in pursuit of a technology's gains. For a technology as uncertain as SB, this may set governments, companies, and other research organizations down vastly differing policy paths and impede consensus to assure security for anyone.

## Increasing monetary and non‐monetary benefits and reducing risks

Many individuals and organizations are actively tackling the biosecurity challenge. The International Genetically Engineered Machine (iGEM) synthetic biology competition, which began in 2004, mandates that organizational leaders and judges conduct rigorous reviews of the materials and planned experiments of each team. Safety and security concerns receive further scrutiny from iGEM's Safety and Security Committee (SSC) and are screened for potential hazards by a commercial partner; all of this is part of the competition's guidance for participating students (Millett *et al*, 2019).

Biosecurity precautions are also incorporated in the “Do‐It‐Yourself” (DIY) biology community's code of ethics in North America and Europe (DIYbio.org), the statement of shared purpose from MIT's Bio Summit 2.0 (www.biosummit.org), the priority of the States Parties to the BWC to establish a code of conduct (Meeting of the States Parties, 2018), the construction of biosecurity norms practices by the African Union for transgenic insects and genetically modified crops (Glover *et al*, 2018), and Australian foreign policy. There is a growing demand for an update to international biosecurity norms and practices akin to the Cartagena Protocol on Biosafety, to increase transparency, cooperation, and collective security in pursuit of SB.

Yet, a question remains of how to invest and incentivize biosecurity with private actors? Such an answer requires focus not only on bench scientists, but also on various gatekeepers, overseers, and watchdogs involved in biotechnology research and development (e.g., the World Organisation for Animal Health's Guideline for Responsible Conduct in Veterinary Research). For example, the furtherance of dual‐use research might be better controlled by training journal editors on what constitutes a potential information hazard within article submissions. Such considerations extend to the grant review process, where funders can require an up‐to‐date understanding of possible information and security hazards that may ensue over the course of the proposed work. In these and other instances, top‐down and bottom‐up collaboration is necessary to raise biosecurity awareness and to identify security threats, while bottom‐up organizations, agencies, and universities conduct on‐the‐ground passive surveillance of possible dual‐use security threats.

One example of this fusion includes the US Federal Bureau of Investigation (FBI), which has sponsored and been working with iGEM to increase awareness of risks and to gain an understanding of possible or developing threats. Though no biosecurity effort will eliminate all threats—nor is such an environment desirable if it means universally forbidding research and innovation that can greatly benefit society—a layering of strengths and capabilities by government and private institutions will provide a more unified effort for biosecurity and might disincentivize actors from leveraging gaps in oversight to develop a biological weapon.

## Conclusions

SB is a transformative technology poised to have at least as much impact as the digital revolution. As with scientific breakthroughs of the past two centuries, the potential for its misuse is globally present and warrants scrutiny at the highest levels of policy discourse. While some protection may be provided by developing specific countermeasures, preventative action may be more reliable. Biosecurity policies and practices must be updated to accommodate the novel challenges associated with SB and acknowledge the globalized and diverse nature of its threat space.

Effective global biosecurity will not happen quickly nor will it be enthusiastically adopted by all governments or non‐governmental organizations. Incentives to misuse synthetic biology with harmful consequences remain high for certain negligent actors, and the coming years may see such events affecting human, animal, or environmental health. Successful biosecurity implementation must be adaptable to quickly incorporate uncertainty as well as new capabilities. Urgent steps are required to place such notions into practice before a major threat incident, both to prevent the damage and subsequent policy reactions that could limit or ban technology platforms entirely. Now is the time to take steps to apply biosecurity to maximize technological benefits while minimizing its dual‐use potential by improving the framing, prioritization, and governance of biosecurity risks.

## Disclaimer

The statements herein are the author's opinions only and not necessarily representative of their host institutions.

## Conflict of interest

The authors declare that they have no conflict of interest.

**Box 1: Further Reading**

**Security Policy and Governance: National and Academic Perspectives**

Chinese Academy of Sciences (2018) *China to have professional committee of synthetic biology*. http://english.cas.cn/newsroom/archive/news_archive/nu2018/201811/t20181115_201249.shtml

Dixon T (2019) Mapping the potential impact of synthetic biology on Australian foreign policy. *Austr J Int Aff* 73: 270–288

Gronvall GK (2018) Safety, security, and serving the public interest in synthetic biology. *J Ind Microbiol Biotechnol* 45: 463–466

Meeting of the States Parties (2018) *Meeting of experts on review of developments in the field of science and technology related to the convention: reflections and proposals for possible outcomes submitted to the meeting of the states parties*. Geneva, Switzerland

Nielsen J, Archer J, Essack M, Bajic VB, Gojobori T, Mijakovic I (2017) Building a bio‐based industry in the Middle East through harnessing the potential of the Red Sea biodiversity. *Appl Microbiol Biotechnol* 101, 4837–4851

World Economic Forum (2020) *Biosecurity innovation and risk reduction: a global framework for accessible, safe and secure dna synthesis*. http://www3.weforum.org/docs/WEF_Biosecurity_Innovation_Risk_Reduction.pdf

Responsible Care Programme. https://www.icca-chem.org/responsible-care/

RMSHE (2019) Approval of the federal research programme for genetic technologies development for 2019–2027. Russian Ministry of Science and Higher Education, Government Decision, Moscow, Russia

**Synthetic Biology: Science, risks and benefits**

Ahteensuu M (2017) Synthetic biology, genome editing, and the risk of bioterrorism. *Sci Eng Ethics* 23: 1541–1561

DiEuliis D, Rao V, Billings EA, Meyer CB, Berger K (2019) Biodefense policy analysis—a systems‐based approach. *Health Secur* 17: 83–99

National Academies of Sciences, Medicine (2018) *Biodefense in the age of synthetic biology*. National Academies Press. https://doi.org/10.17226/24890

van der Vlugt CJ, Brown DD, Lehmann K, Leunda A, Willemarck N (2018) A framework for the risk assessment and management of gene drive technology in contained use. *Appl Biosaf* 23: 25–31

**Synthetic Biology and Society**

Normile D (2018) *Shock greets claim of CRISPR‐edited babies*. American Association for the Advancement of Science. https://doi.org/10.1126/science.362.6418.978

Trump BD, Cegan JC, Wells E, Keisler J, Linkov I (2018) A critical juncture for synthetic biology. *EMBO Rep* 19. https://doi.org/10.15252/embr.201846153

National Academies of Sciences, Engineering, and Medicine (2020) *Safeguarding the bioeconomy*. National Academies Press.

World Organization for Animal Health (2019) *Guidelines for responsible conduct in veterinary research*. https://www.oie.int/fileadmin/Home/eng/Our_scientific_expertise/docs/pdf/BTR/A_GUIDELINES_VETERINARY_RESEARCH.pdf
